# Predictors of Quality-of-Life Improvement at Different Minimum Clinically Important Difference Values in Patients with Chronic Obstructive Pulmonary Disease after Climatic Rehabilitation Treatment

**DOI:** 10.3390/life13081763

**Published:** 2023-08-17

**Authors:** Anna Kubincová, Peter Takáč, Lucia Demjanovič Kendrová, Pavol Joppa

**Affiliations:** 1 Department of Physical Medicine, Balneology and Medical Rehabilitation, Medical Faculty of P. J. Šafárik University and L. Pasteur University Hospital in Košice, 04190 Košice, Slovakia; anna.kubincova@upjs.sk; 2 Department of Physiotherapy, Faculty of Health Care, University of Prešov, 08001 Prešov, Slovakia; lucia.kendrova@unipo.sk; 3 Department of Pneumology and Phtiseology, Medical Faculty of P. J. Šafárik University and L. Pasteur University Hospital in Košice, 04190 Košice, Slovakia; pavol.joppa@upjs.sk

**Keywords:** pulmonary rehabilitation, COPD, minimum clinically important difference (MCID) quality of life (SGRQ), climatic rehabilitation, climatotherapy

## Abstract

Background: The minimum clinically important difference (MCID) for the St George’s Respiratory Questionnaire (SGRQ) is debated in chronic obstructive pulmonary disease (COPD) quality-of-life (QoL) assessments. This study aimed to determine whether there is a difference in predictors of clinically significant improvement between the traditional (value of 4) and newly proposed MCID SGRQ (value of 7) after climatic rehabilitation treatment. Climatic rehabilitation treatment consists of two main parts: climatotherapy, which typically involves the controlled exposure of individuals to natural environmental elements, and climatic rehabilitation, which includes other therapeutic factors such as physical activities as well as educating the patient to change their lifestyle. Methods: This study included 90 consecutive patients diagnosed with COPD who underwent structured complex pulmonary rehabilitation in High Tatras, part of the Carpathian Mountains. The examination before and after treatment included spirometry, QoL assessment using the SGRQ, 6 min walk test (6-MWT), and the Borg, Beck and Zung scale. Results: Patients showed statistically significant improvement after the intervention in FEV1, FEV1/FVC, 6-MWT, (*p* < 0.001), anxiety scores, depression, and improvement in dyspnoea both before and after the 6-MWT (*p* < 0.001). For both MCID for SGRQ levels 4 and 7, we confirmed the same predictors of clinical improvement for bronchial obstruction grade (spirometry) and exercise capacity (6-MWT), for quality of life in activity score and total score. Conclusion. The results suggest that both the proposed MCID for SGRQ values could be sufficient to assess the clinical significance of the achieved change in health status when assessing the need for pulmonary rehabilitation comprising climatotherapy in patients with COPD.

## 1. Introduction

Chronic obstructive pulmonary disease (COPD) is one of the most common causes of death worldwide and has a complex impact on patients [1], including quality of life (HRQL) [2,3,4,5,6,7]. The lives of people with chronic diseases change dramatically, leading to significant deterioration in their quality of life. According to Rozinczuk et al. [1], in patients with COPD, age, shortness of breath, longer disease duration, comorbidities, oxygen therapy, and family burden of respiratory disease reduce their quality of life (QOL) [1]. The consequences of chronic respiratory diseases, including COPD, include peripheral muscle impairment, respiratory muscle impairment, nutritional impairment, cardiac function, skeletal impairment, sensory deficits, and psychosocial dysfunction. These systemic impairments magnify the impact of the disease on the quality of life [2].

In 2021, according to the National Centre for Health Information, there were 68,242 patients with COPD in Slovakia [3]. There is relevant evidence regarding the importance of pulmonary rehabilitation [4,5,6,7]. Gutenbrunner et al. [8] proposed a definition of spa medicine and hydrotherapy, according to which, “Spa medicine includes” all medical activities that originated and are derived in spas on the basis of scientific knowledge and are aimed at health promotion, prevention, therapy and rehabilitation”. Basic elements of spa interventions include balneotherapy, hydrotherapy, and climatotherapy [8,9,10]. In addition, more advanced environmental modification strategies have been proposed, such as “health resort medical rehabilitation (HRMR)” and “high-altitude climatotherapy (HACT, also known as “alpine” therapy” [9]. Spa medicine is also recommended by the World Health Organization (WHO) strategy for non-communicable diseases (NCDs) and universal health coverage [11]. A meta-analysis of studies of mountain climate therapy (HACT) for asthma confirmed its efficacy in improving lung function [9,12]. According to the current pilot research study conducted by Gvozdjakova et al. in 2022 [13], pulmonary rehabilitation in a mountain environment may improve platelet mitochondrial bioenergetics, improve functional capacity (dyspnoea), and accelerate patient recovery. A previous study showed that platelet mitochondrial respiration parameters were improved in patients with post-COVID-19 syndrome following this rehabilitation method [13]. Similarly, in an experimental study involving climate therapy, improvements in mitochondrial complex I (CI) capacity parameters associated with oxidative phosphorylation (OXPHOS) and electron transfer (ET) in platelets were found [14]. Climatic treatment facilities are usually established in areas with a local climate that is suitable for the treatment of lung diseases. For a place to be recognised as a climatic site, it is necessary to demonstrate healing properties based not only on medical experience but also on scientific research on the properties of climate and its biological effects [15]. QOL is an important criterion for evaluating treatment efficacy in comprehensive treatment and pulmonary rehabilitation of COPD [16]. Both general and specific tools are used to measure QOL in patients [17,18,19]. The Saint George Specific Respiratory Questionnaire (SGRQ) has been used since 1991 to assess disease-specific health status in COPD patients [20], and changes in scores have been used to evaluate the effectiveness of interventions, including pulmonary rehabilitation [21].

To assess the clinical significance of the change in health status, the minimum clinically important difference (MCID) was used to quantify when the measured differences could be considered clinically relevant. It has been defined as the smallest difference in scores in the domain of interest that patients consider beneficial [22,23].

The MCID is suitable for assessing the effects of pulmonary rehabilitation on the quality of life. For the MCID for SGRQ, several older studies assessing changes in SGRQ quality of life have used a standard value of four units [24,25]. In the Cochrane review, investigators pooled three separate scales (St George’s Respiratory Questionnaire (SGRQ), Chronic Respiratory Disease Questionnaire (CRQ), and COPD Assessment Test (CAT)), which assessed HRQoL in a meta-analysis. All the scales revealed a better improvement in quality of life with inspiratory muscle training, but unlike CRQ and CAT, only the lower limit of the 95% CI of SGRQ exceeded the MCID (−4 units; very low-certainty evidence). They noticed a larger effect estimate favouring the intervention in some items when other scales were used (CAT, SF-36, CCQ) [26]. However, several recent studies have questioned this threshold and have suggested an MCID SGRQ of 7. The authors of these studies performed statistical analyses based on anchoring and distribution, evaluating the degree of overlap of confidence intervals and the use of triangulation [23,27,28,29]. Therefore, we took advantage of these findings and focused on evaluating the predictors of MCID SGRQ improvement at both levels.

## 2. Aim of the Research

To demonstrate the applicability of these findings in clinical practice, we sought to determine which of the clinical baseline parameters that exhibited enhancement after climatic rehabilitation treatment (also known as climatotherapy-rehabilitation) were predictive of clinically significant improvement in SGRQ quality of life in both the traditional level 4 MCID and the new level 7 MCID.

## 3. Materials and Methods

### 3.1. Patients

The follow-up involved patients with COPD who underwent complex climatic and rehabilitation treatments in climatic spas in High Tatras. The spas (at four different locations) are located at altitudes ranging from 760 to 1067 m above sea level, corresponding to alpine mountain altitudes. The diagnosis of COPD was based on the post-bronchodilator ratio of forced expiratory volume in 1 s to forced vital capacity (FEV1/FVC), which was <0.7 [30].

Exclusion criteria for inclusion in the climatic rehabilitation treatment were acute exacerbation of COPD, respiratory insufficiency, ischaemic heart disease (all stages according to NYHA), myocardial infarction, or stroke in the history. Non-cooperation or non-compliance of patients and significant anxiety or depression were also a contraindication. The study was approved by the Research Ethics Committee. (Ethic Commission of the University Prešov in Prešov at the session of 18 November 2015). Written informed consent was obtained from all patients.

### 3.2. Characteristics of Treatment

The climatic rehabilitation stays in the mountain spa lasted for 3 weeks. All patients underwent a standard program, based on exercise training according to international recommendations [7,31]. The program also included respiratory physiotherapy and physical therapy including hydrotherapy, strength training, and climatotherapy. Exercise training was prescribed individually according to the objective assessment of the patient and, if well tolerated, had an increasing tendency. The intensity of the training was monitored according to the Borg scale and set to grade 3. Patients followed the program daily, except for weekends. The structure of the rehabilitation program has been described in detail in our previous publication [32].

### 3.3. Measurements

The patients were examined at the beginning and end of the climatic—rehabilitation intervention.

At baseline, examinations included spirometry (FEV1 and FEV1/FVC), 6 min walk test (6-MWT), and assessment of dyspnoea using the Borg Stress Scale and the Modified Medical Research Council Dyspnoea Scale (mMRC), Measurement of impact on COPD patient’s well-being and daily life CAT (COPD Assessment Test), anxiety (Beck Anxiety Inventory) depression (Zung Self-Rating Depression Scale), and QoL assessment SGRQ (St George’s Respiratory Questionnaire). We used the mMRC and CAT because these questionnaires are usually used for the ABCD classification of COPD according to GOLD [30]. At the end of the climatic rehabilitation intervention, examinations included spirometry (FEV1 and FEV1/FVC), 6 min walk test (6-MWT), Borg Exercise Scale, depression, anxiety, and QoL assessment. The 6-MWT was used to assess locomotor performance. This test is widely used to assess the effects of treatment in individuals with various cardiovascular and pulmonary diseases including COPD. The 6-MWT was performed indoors along a corridor (30 m) in accordance with international recommendations as described in our previous publication [33]. The patient-completed CAT questionnaire assesses the global impact of COPD (cough, sputum, shortness of breath, chest distress) on the quality of life (health status) [30,33]. The mMRC questionnaire is a modified dyspnoea scale [34] that classifies patients into five grades according to the severity of dyspnoea [16,30]. The Beck Anxiety Inventory is a self-report questionnaire measuring symptoms of anxiety and Zung’s Self-rating Depression Scale (SDS) used to identify the presence of depressive disorders in adults [35,36,37]. The subjective dyspnoea score was evaluated using a modified scale according to Borg [38]. We evaluated QoL using the Saint George’s Specific Respiratory Questionnaire (SGRQ). The SGRQ is a validated questionnaire that measures the quality of life of patients with COPD [39]. It has a total score and three scores for: symptoms, activity, and impact of the disease; each score ranges from 0 (no impairment) to 100 (worst possible) [25,40]. The questionnaire calculated three components: symptoms, activity, impacts, and total scores [20]. We defined the minimum clinically important difference (MCID) of the SGRQ quality of life in chronic obstructive pulmonary disease according to Jones [25], where the summary score is the value of Δ4(Δ change). In addition, we used the MCID SGRQ for the summary score of Δ7(Δ change), according to several studies [23,27,28,29]. We considered the above-cited studies to be sufficiently relevant and, therefore, did not perform the above statistical analyses but considered them as initial findings.

### 3.4. Statistical Analysis

Statistical analysis was performed using the IBM SPSS 19 software. The normality of the data distribution was tested using the Kolmogorov–Smirnov test for each measured parameter. Data are presented as the mean ±1 standard deviation (SD), median (interquartile range), or as percentages, as appropriate. In patients with COPD, the baseline measurements of pulmonary function, 6-MWT distance, anxiety, depression, dyspnoea, and SGRQ scores were compared with the measurements after the intervention of 3 weeks of pulmonary rehabilitation in a mountain environment using the Wilcoxon Signed Rank test. Logistic regression models were used to identify potential predictors of improvement in the Total SGRQ score using MCID of −4 and −7 points, respectively. Parameters that were predictors of improvement in univariate models for each MCID cut-off were further tested in multivariate logistic regression models with age, sex, and BMI as potential confounders. Odds ratios (OR) with corresponding 95% confidence intervals (Cis) are reported for each independent predictor. Statistical significance was set at *p* < 0.05.

## 4. Results

### 4.1. Evaluation of the Impact of Climatic Rehabilitation Treatment

The COPD cohort consisted of 90 patients, 64 (71%) males and 26 (29%) females, with a mean age of 65.7 (SD ± 11.9) years, and a mean BMI of 26.99 (SD ± 5.029). Patients were classified according to GOLD stages of severity of lung function decline as follows: 14 (16%), stage I; 45 (50%), stage II; 27 (30%), stage III; and 4 (4%), stage IV. At the personal examination, we also ascertained whether the respondents had smoked or had smoked in the past and how many cigarettes they had smoked per day. We found that 46 respondents (51%) were non-smokers. Thirty-four (38%) respondents smoked in the past, and 10 (11%) continued to smoke during treatment. This is a research cohort of patients that we have been following in other contexts for a long period of time [32]. According to the modified dyspnoea rating scale (mMRC), the patients had a mean grade of 2.04 (SD ± 0.85). The distribution was as follows: grade 1, 28 (31.1%); grade 2, 32 (35.6%); grade 3, 28 (31.1%); and grade 4, two (2.2%). The mean CAT questionnaire score was 15.08 (SD ± 6.80), indicating impaired general health. All patients with COPD experienced statistically significant improvement after the intervention with climatic rehabilitation treatment for objective measures of ventilatory function in FEV1, FEV1/FVC, and exercise capacity assessed using the 6-MWT (*p* < 0.001 for all parameters). There was also a statistically significant improvement in the observed individual and summate (Total) SGRQ QoL scores as well as in the other subjective parameters studied, including dyspnoea before and after the 6 min walk test, Beck anxiety score, and Zung depression score (*p* < 0.001 for all parameters, Table 1.

The MCID for SGRQ Total score was <4 in 17 patients (18.9%), ≥4 in 73 patients (81.1%), <7 in 25 patients (27.8%), and ≥7 in 65 patients (78.2%). The Δ MCID for SGRQ values before and after climatic rehabilitation treatment are shown in Table 2.

### 4.2. Predictors of MCID Quality-of-Life SGRQ

Predictors of MCID in SGRQ quality-of-life total score Δ SGRQ 4 and Δ SGRQ 7 after pulmonary rehabilitation in patients with CODP. The mean MCID Δ SGRQs in our study were for Symptoms score −20, Activity score −12.7, Impacts score −7.3 and Total Score −15.9.

A clinically significant improvement in the quality-of-life SGRQ at Total Score of four units was observed in 73 patients, that is, 81.1% with COPD. Predictors of clinically significant improvement in quality of life were lower baseline pulmonary function test scores (FEV1/FVC) even after correcting for age, sex, and BMI (*p* = 0.001). Another predictor was a lower baseline distance achieved in the 6 min walk test (*p* = 0.001), even after correcting for age, sex, and BMI (*p* = 0.001). Among the subjective parameters, higher CAT scores (*p* = 0.021), higher activity scores (*p* < 0.001), and higher total scores (*p* = 0.03) were predictors of SGRQ quality-of-life domains, even after adjusting for age, sex, and BMI (*p* < 0.05). A clinically significant improvement in SGRQ quality of life at a total MCID score of seven units was observed in 65 patients (72.2%) with COPD. The predictors of clinically significant improvement in SGRQ quality of life in patients with COPD were lower entry FEV1% (*p* = 0.041) and lower entry distance achieved in the 6 min walk test (*p* = 0.040) after adjusting for age, sex, and BMI (*p* < 0.05). For quality-of-life domains, SGRQ predictors were higher input domain Symptoms score (*p* = 0.019), higher input domain Activity score (*p* = 0.002), and higher input domain Total score (*p* = 0.008), all even after adjustment for age, gender representation, and BMI (*p* < 0.05), Table 3. For both Δ SGRQ 4 and Δ SGRQ 7 levels of clinically significant improvement (MCID) in SGRQ quality of life, the objective predictors were the same: the 6-MWT input values and the degree of bronchial obstruction; the worse the input values, the more significant the clinical improvement of the patients. For both MCID clinical improvement values Δ ΔSGRQ 4 and ΔSGRQ 7, the predictors of improvement in subjective parameters from the subdomains were the SGRQ activity score, which refers to activities limited by shortness of breath; the more pronounced the patient’s sensation of shortness of breath at the start of treatment, the more pronounced the improvement occurred. Similarly, a worse SGRQ quality-of-life summary score at the start of treatment was a predictor of clinical improvement in MCID ΔSGRQ 4 and ΔSGRQ 7. Among the other parameters, for the MCID SGRQ 4 units, the predictor of quality-of-life improvement was the baseline CAT score, and for the MCID SGRQ 7 units, the predictor of clinical improvement in quality of life was the baseline SGRQ symptoms score.

Subjective anxiety and depression were not identified as predictors of improvement despite their frequent use in various COPD PR research papers. Similarly, the SGRQ Impact score, which reflects aspects related to social function and psychological disturbances resulting from respiratory diseases, was not a predictor.

## 5. Discussion

COPD is not only one of the leading causes of death; because it often causes numerous systemic impairments, it has a significant negative impact on the quality of life. Pulmonary rehabilitation is now an integral part of the comprehensive management of patients with COPD based on several international guidelines [2,9,10,11]. Climatotherapy or health resort medical rehabilitation (HRMR) and high-altitude climate therapy have long been integral parts of complex rehabilitation in some countries. These modalities have been introduced into medical practice largely on the basis of empirical findings as well as assumptions about the beneficial effects of environmental modification; despite this, up to this time we have no satisfactory experience and scientific information about the influence of environmental and climatic conditions on human health [12]. Therefore, researchers are looking for as much experimental [11,13,14] and clinical evidence [15,32] as possible on the effectiveness of this therapy. This study aimed to determine whether there is a difference in the predictors of clinically significant improvement between the traditional (value of 4) and newly proposed MCID SGRQ (value of 7) after climatic rehabilitation treatment. Saint George’s Specific Respiratory Questionnaire (SGRQ) has been used since 1991 to assess disease-specific health status in patients with COPD [23]. Clinically Important Difference (MCID) is now commonly used in research to determine whether statistically observed changes can be considered clinically significant. It is similar to that of MCID in the SGRQ, where a four-unit level was previously proposed [27,28]. The MCID for the SGRQ was thoroughly analysed. Three fundamental methodologies were used: patient judgement, clinician judgement, and criterion referencing. The methodology as well as the issues of creating different MCID values for the SGRQ are discussed in more detail, and the advantages and disadvantages of each of these approaches are evaluated. We consider as an important remark the statement of some experts: “Whilst many treatment studies compare treatments by estimating the mean difference, there is a significant disadvantage to this approach, not least because there is a risk that if the mean difference is <4 units, the treatment may be judged to be ineffective. However, for the mean difference to exceed 4.0, more than half of the patients would need to improve by ≥4 units (if the data were normally distributed). This is a very high threshold for judging the efficacy of a treatment” [41]. Because of the difficulties in the clinical interpretation of the SGRQ quality of life, we decided to investigate which observed clinical baseline parameters with documented improvement after climatic rehabilitation treatment appeared to be crucial for the clinical impact of the disease-minimal clinical difference (MCID). All COPD patients experienced statistically significant improvement after the intervention with climatic rehabilitation treatment for objective measures of ventilatory function in FEV1, FEV1/FVC, and exercise capacity assessed using the 6-MWT (*p* < 0.001 for all parameters). There was also a statistically significant improvement in the observed individual and summated SGRQ quality-of-life scores as well as in the other subjective parameters studied, including dyspnoea before and after the 6 min walk test, Beck anxiety score, and Zung depression score (*p* < 0.001 for all parameters). We consider our most important findings as follows:

For both Δ SGRQ 4 and Δ SGRQ 7 levels of clinically significant improvement (MCID) in SGRQ quality of life, the objective predictors were the same: the 6-MWT input values and the degree of bronchial obstruction; the worse the input values, the more significant the clinical improvement of the patients. For both MCID clinical improvement values Δ ΔSGRQ 4 and ΔSGRQ 7, the predictors of improvement in subjective parameters from the subdomains were the SGRQ activity scores, which refer to activities limited by shortness of breath; the more pronounced the patients’ sensation of shortness of breath at the start of treatment, the more pronounced the improvement occurred. Likewise, a worse SGRQ quality-of-life summary score at the start of treatment was a predictor of clinical improvement in MCID ΔSGRQ 4 and ΔSGRQ 7. In the study of Alma et al. [26], the baseline and follow-up data in the St. George’s Rating Questionnaire (SGRQ) were retrospectively analysed from pulmonary rehabilitation (PR) and routine clinical practice (RCP). The MCID SGRQ estimates of improvement for COPD Stage I and II were around −9.90 (−11.28 to −8.52) for COPD Stage III and IV. −6.64 (−8.15 to −5.12). In a more recent publication of studies by Hassali et al. [42] using the anchor-based approach and the distribution-based approach, the MCID value was calculated as 5.07 (95% CI—2.54–12.67) and 6.05 (5.30–6.80). The determined MCIDs were comparable to those reported by the Chinese version of the SGRQ (MCID 6.6 (95% CI–0.8–14.1)) [43]. In both studies, the MCID value was higher than the MCID value recommended by Jones (2014), that is, four units [16]. Hassali et al. [42] reported that the difference in values may be due to differences in the populations studied and also the difference in cultures of Asian and European populations. According to the results of the research of Harm Alma, 2016 [27], a clinically significant improvement in the MCID SGRQ must exceed a value of 7 in the SGRQ summary score. This large difference between the original value and new research may have been influenced by differences in the study setting, age of the patients, time period of measurement, and various health status criteria. Another explanation may be the poor methodological quality of the patient-centred approach. In our study, the difference in pre- and post-intervention total scores was Δ −15.9 ± 1.68. Similar findings were reported in the research of Alma et al. [28], where MCID SGRQ was tracked over longer periods of time after pulmonary rehabilitation. The MCID estimates in clinical improvement ranged from −10.3 to −7.6 for MCID total score SGRQ. The use of very low MCID SGRQs reported in older publications may lead to an overestimation of the interpretation of treatment effects in patients with COPD. Climatic rehabilitation facilities are used to varying extents in different countries, mainly owing to different climatic conditions. In some countries, these are not classified as health facilities. Moreover, even in countries where their use has a rich tradition, these facilities have different positions in the health care system. Health insurance companies’ level of financial coverage also varies. In our view, research that provides insights into climatic rehabilitation from different perspectives is particularly important. The expected benefits of this knowledge would be to optimise the indications for lung disease therapy in climatic rehabilitation, patient selection, and the unification of effective treatment protocols at different stages of the disease.

## 6. Limitations

Our patient sample included patients with different COPD stages. We hypothesised that predictors for different stages may differ; however, the research aim was to find the predictors for the two MCID values in the same research sample. A relatively low sample size together with a low number of patients in the advanced disease severity stages GOLD 3 and 4 represent another weakness of our study. We also consider it a weakness that subjects may include those with potential comorbidities such as interstitial lung disease and pulmonary hypertension because chest computed tomography (CT), echocardiography, and right heart catheterisation were not performed because of the characteristics of the treatment facility. Additionally, it is important to remember that we cannot rule out the potential overlap between asthma and COPD (ACO). One must be cautious regarding the interpretation of our results, notably for more advanced COPD severity stages, and further studies with larger cohorts are warranted.

## 7. Strengths

The strength of our study is the evaluation of pulmonary rehabilitation during climatic rehabilitation treatment in a mountain setting. This is a less-established rehabilitation method that requires as many studies as possible to yield new insights. Subjective and objective parameters that are commonly used in practice were included in this study. Therefore, we believe that the results of this research will provide useful knowledge for clinical practice.

## 8. Conclusions

We found that the input values of the clinical parameters of bronchial obstruction degree and exercise capacity as predictors of clinical improvement in quality of life at the MCID for SGRQ level were four units and seven units, respectively. The predictors of quality of life at the MCID for SGRQ level of four units were the input parameters of CAT score and of other subjective parameters, and at the MCID for SGRQ level of both four and seven units, they were the input parameters of Activity Score and Total Score SGRQ. For the MCID for SGRQ 7 units, the predictor of clinical improvement in quality of life was the input SGRQ Symptoms score. We conclude that even the MCID for SGRQ level 7 proposed in recent studies could be introduced into clinical practice to assess the clinical significance of the health status change achieved. Similarly, the SGRQ Impacts Score, which reflects aspects related to social function and psychological disturbances resulting from respiratory disease, was not a predictor. These data expand our knowledge on climatic rehabilitation treatments. These subjective parameters require further research, and one possible explanation is the time interval of the therapeutic intervention.

## Figures and Tables

**Table 1:** Median values and statistical comparison of the group of COPD patients before and after climatic rehabilitation treatment in objective and subjective parameters.

Parameter	Before Intervention	After Intervention	Z-Value	*p*
	Median (Interquartile Range)			
FEV_1_ (% predicted)	58.0 (44.1–70.8)	60.0 (45.0–76.0)	7.84	<0.001
FEV_1_ (ml)	1623.9 (1133.4–2020.8)	1743.0 (1229.2–2065.0)	7.82	<0.001
FEV_1_/FVC (%)	51.8 (50.0–60.9)	57.0 (48.1–66.7)	6.96	<0.001
Beck score	13.5 (7.0–20.0)	7.0 (5.0–14.0)	−8.06	<0.001
Zung score	52.0 (46.0–59.0)	48.0 (44.0–51.0)	−7.34	<0.001
6-MWT (m)	220.0 (180.0–360.0)	250.0 (200.0–400.0)	7.99	<0.001
Borg before walking	1.0 (0.0–2.0)	1.0 (0.0–2.0)	−4.58	<0.001
Borg after walking	3.0 (3.0–5.0)	3.0 (2.0–4.0)	−4.98	<0.001
SGRQ				
Symptom score	59.7 (43.3–69.6)	39.7 (29.3–52.6)	−7.62	<0.001
Activity score	66.2 (53.5–79.0)	53.5 (41.4–60.3)	−7.67	<0.001
Impact score	34.8 (26.8–52.4)	27.5 (15.4–41.0)	−6.68	<0.001
Total Score	51.8 (39.0–61.5)	35.9 (27.2–47.6)	−7.95	<0.001

6-MWT—six-minute walking test, FEV1—forced expiratory volume in one second, FVC—forced vital capacity, SGRQ—St. George’s Respiratory Questionnaire.

**Table 2:** MCID SGRQ before and after climatic rehabilitation treatment.

MCID SGRQ	Δ
Symptom score	−20.0 ± 2.11
Activity score	−12.7 ± 2.66
Impact score	−7.3 ± 1.8
Total score	−15.9 ± 1.68

Δ—change.

**Table 3:** Predictors of clinically important improvement (MCID) in SGRQ quality of life Total score Δ SGRQ 4 and Δ SGRQ 7 after the pulmonary rehabilitation in CODP patients.

Parameter at Baseline	Crude OR ΔSGRQ 4	95% CI	*p*	Adjusted * OR ΔSGRQ 4	95% CI	*p*	Crude OR Δ SGRQ 7	95% CI	*p*	Adjusted * OR Δ SGRQ 7	95% CI	*p*
FEV_1_ (%)	0.981	(0.959–1.003)	0.092	N/A			0.979	(0.959–0.999)	0.041	0.969	(0.946–0.994)	0.014
FEV_l_ mL	0.998	(0.998–1.000)	0.083	N/A			0.999	(0.998–1.000)	0.056	N/A		
FEV_1_/FVC (%)	0.921	(0.868–0.977)	0.007	0.871	(0.802–0.947)	0.001	0.968	(0.927–1.012)	0.152	N/A		
CAT score	1.108	(1.013–1.211)	0.025	1.123	(1.017–1.239)	0.021	1.073	(0.997–1.155)	0.061	N/A		
mMRC score	1.875	(0.946–3.716)	0.072	N/A			1.181	(0.679–2.052)	0.556	N/A		
Beck score	0.975	(0.916–1.038)	0.429	N/A			0.995	(0.940–1.052)	0.847	N/A		
Zung score	1.007	(0.954–1.062)	0.811	N/A			1.026	(0.977–1.077)	0.308	N/A		
6-MWT (m)	0.991	(0.986–0.996)	0.001	0.991	(0.985–0.996)	0.001	0.995	(0.991–1.000)	0.036	0.995	(0.991–1.000)	0.040
Borg before walking	1.432	(0.860–2.384)	0.167	N/A			1.122	(0.741–1.700)	0.586	N/A		
Borg after walking	1.136	(0.844–1.528)	0.399	N/A			1.002	(0.785–1.278)	0.989	N/A		
Symptoms score	1.024	(0.997–1.051)	0.087	N/A			1.026	(1.002–1.051)	0.037	1.032	(1.005–1.060)	0.019
Activity score	1.067	(1.026–1.109)	0.001	1.077	(1.033–1.122)	0.000	1.049	(1.016–1.082)	0.003	1.054	(1.020–1.090)	0.002
Impacts score	1.024	(0.994–1.055	0.123	N/A			1.018	(0.993–1.044)	0.166	N/A		
Total score	1.047	(1.010–1.085	0.012	1.066	(1.022–1.112	0.003	1.037	(1.006–1.070)	0.020	1.048	(1.012–1.084)	0.008

6-MWT—six-minute walking test, CAT—COPD Assessment Test, CI—confidence interval, FEV1—forced expiratory volume in one second, FVC—forced vital capacity, mMRC—modified Medical Research Council dyspnoea scale, OR—odds ratio, SGRQ—St. George’s Respiratory Questionnaire. *—adjustment for age, sex, and body mass index.

## Data Availability

Data presented in this study are available on request from the corresponding author. The data are not publicly available due to privacy restrictions.
